# Giant True Celiac Artery Aneurysm

**DOI:** 10.4103/1319-3767.45056

**Published:** 2009-01

**Authors:** Badr Aljabri

**Affiliations:** Department of Surgery, King Khalid University Hospital, King Saud University, Riyadh, Saudi Arabia

**Keywords:** Celiac artery aneurysm, splanchnic artery aneurysm, rupture, young women

## Abstract

Celiac artery aneurysms are rare and usually asymptomatic. The management of these aneurysms is challenging, especially when they are large and involve the confluence of the trifurcation. We present here a case of a large celiac artery aneurysm involving its branches in a young woman. Preoperative investigations, intraoperative findings, and the operative procedure are also presented and discussed.

Celiac artery aneurysm is a rare type of splanchnic artery aneurysm. They are usually asymptomatic and discovered incidentally during diagnostic imaging. Large symptomatic celiac artery aneurysms are extremely rare and warrant an urgent intervention to prevent rupture and death. We present here a case of a large, symptomatic celiac aneurysm in a young female patient, who was treated surgically without hepatic artery revascularization.

## CASE REPORT

A 23-year-old woman, a mother of two healthy kids, presented with a tender, pulsatile mass located in the epigastric area. The mass was discovered four months prior to hospital admission when she had frequent episodes of abdominal pain without any other gastrointestinal symptoms. Additionally, there was no history of abdominal trauma or other symptoms that were suggestive of connective tissue disease. She had no history of oral or genital ulcers, and her medical and family history was unremarkable.

On physical examination, the patient appeared healthy and not in distress. Her vital signs were stable, and peripheral pulses were normal. There was a large, tender, pulsatile, epigastric mass, and the rest of the abdominal examination was unremarkable.

**Figure 1:**
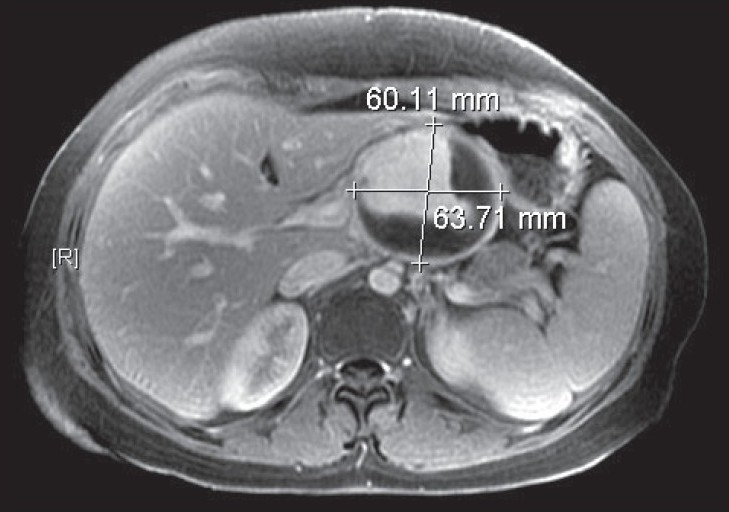
Magnetic resonance imaging of the abdomen demonstrates a large celiac artery aneurysm involving the trifurcation

**Figure 2:**
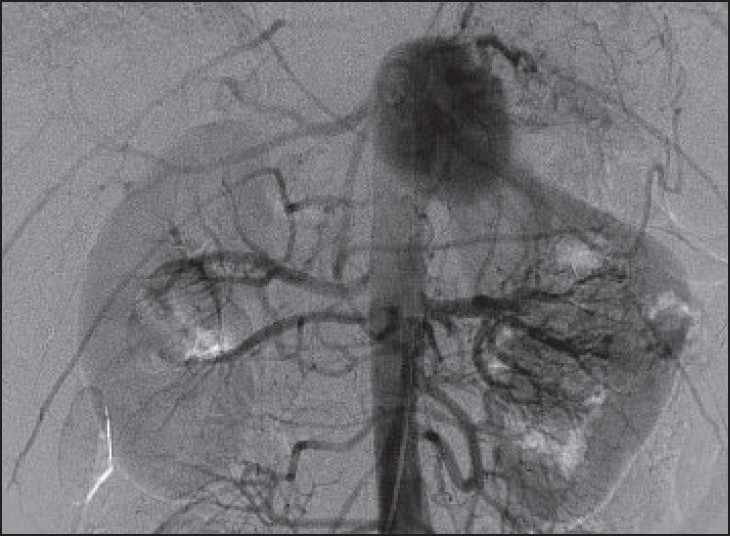
Aortography demonstrates a celiac artery aneurysm originating from its trifurcation with a patent gastroduodenal artery

Her routine blood investigations, C-reactive protein, and erythrocyte sedimentation rate were within normal limits. Her connective tissue disease workup, including antinuclear antibodies, was negative. Abdominal and thoracic magnetic resonance imaging was performed, which revealed a large, epigastric, arterial aneurysm with an intramural thrombus measuring 6.3 × 6.0 cm in its maximum transverse diameters. This thrombus originated from the confluence of the celiac artery trifurcation with a nonenhancing wall, suggestive of degenerative aneurysm and excluding (in conjunction with the connective tissue workup) the possibility of Behcet's disease [Figure 1]. There were no other pathologies identified. Echocardiography showed normal valvular and myocardial functions. To evaluate her mesenteric circulation, a transfemoral aortography was performed with selective angiography of the mesenteric arteries, which showed a normal caliber celiac artery trunk with an aneurysm originating from its trifurcation [Figure 2]. The gastroduodenal artery was patent with good collateral circulation to the superior mesenteric artery.

**Figure 3:**
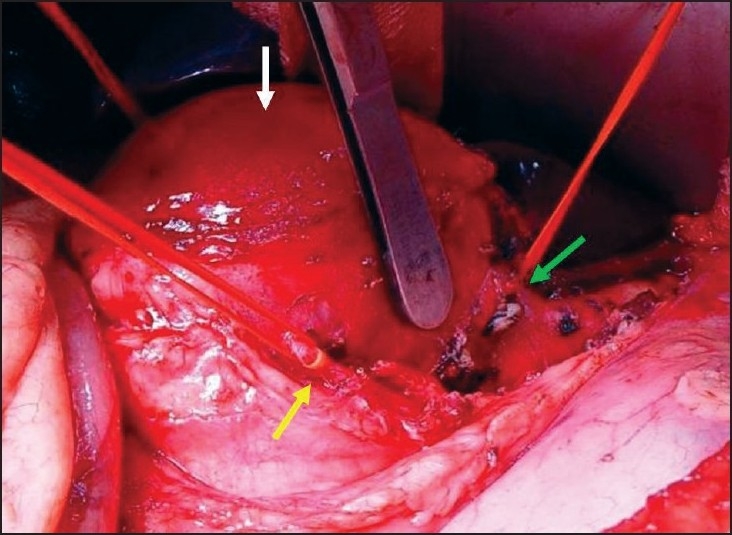
Intraoperative photograph shows the celiac trunk (green arrow), splenic artery (yellow artery), and celiac artery aneurysm (white arrow)

The patient underwent surgical exploration and repair of the celiac artery aneurysm, keeping in mind the possible need for revascularization of the hepatic artery. During surgical exploration, a longitudinal transperitoneal approach was utilized to access the supraceliac part of the abdominal aorta after incising the gastrohepatic ligament and mobilizing the left lobe of the liver to the right. A suitable area for aortic clamping was created and sharp dissection was carried down caudally along the anterior wall of the aorta. The celiac trunk was identified under the aneurysm and controlled. Common hepatic, splenic, and left gastric arteries were also similarly controlled [Figure 3]. An intraoperative duplex ultrasonography was performed at this stage to evaluate the blood flow in the hepatic artery proper after excluding the blood flow in the common hepatic artery. The blood flow was sufficient not to warrant a hepatic artery revascularization. The celiac trunk was ligated along with its trifurcation. Then, the aneurysm cavity was opened, and the intramural thrombus was evacuated. A specimen from the aneurysm wall was sent for histological evaluation, and the aneurysm sac was closed after ensuring hemostasis; the abdominal wall closure proceeded in the usual fashion.

The patient's postoperative course was uneventful, and she was discharged from the hospital on the sixth postoperative day. Histopathological investigation of the aneurysm wall demonstrated a true degenerative aneurysm. Twenty-five months after the operation, the patient was living a normal life with no abdominal symptoms, and her abdominal ultrasound examination of the aorta revealed no abnormalities.

## DISCUSSION

Celiac artery aneurysm is a rare type of splanchnic artery aneurysm that causes a high risk for mortality if they rupture.[1–3] They are usually asymptomatic and are discovered incidentally. Although asymptomatic, the incidence of rupture has been decreasing in the last few decades because of advancements in imaging modalities.

Most of these aneurysms are small during diagnosis, and hence, treatments with endovascular methods are amenable and carry low risk of morbidities.[4,5] Large celiac artery aneurysms involving the confluence of its trifurcation are usually challenging to treat, and surgical intervention carries a 5% mortality risk.[1,6] In our case, the celiac artery aneurysm was extremely large and involved the trifurcation, which was surprising to see in a young lady who did not have any form of connective tissue disease or recent sepsis indicative of mycotic aneurysm. The decision to select surgical intervention was dependent on the fact that coil embolization was clearly an inferior approach because of the large size and the location of the aneurysm, and the fact that the patient was otherwise healthy and young.[2]

It is still under debate as to whether the revascularization of the hepatic artery after aneurysm resection offers any advantage. There is no strong evidence to support either treatment option.[7] Clearly, if gross liver ischemia is encountered after aneurysm resection, revascularization of the hepatic artery is required either by an antegrade supraceliac aortohepatic bypass, or retrograde inflow from the infrarenal segment of the aorta or the common iliac arteries.

However, there are some methods to predict the likelihood of the need for hepatic artery revascularization. The patency and size of the gastrodudenal artery could be evaluated in preoperative angiography and an intraoperative duplex scan could be used (as in our case, which has not been reported previously) to evaluate the blood flow velocity in the hepatic artery proper, after occluding the common hepatic artery. Patent gastrodudenal artery with retrograde contrast reflux into the branches of the superior mesenteric artery and triphasic waves at the hepatic artery by duplex interrogation are signs that suggest that revascularization of the hepatic artery is not required.

In the present case, the celiac artery aneurysm was resected without reconstruction because of angiographic evidence that the gastroduodenal artery was patent and large, and intraoperative evidence of adequate flow in the hepatic artery, as documented by duplex ultrasonography.
